# Racial and Ethnic Disparities in the Management and Outcomes of Acute Myocardial Infarction Complicated by Ventricular Arrhythmias

**DOI:** 10.3390/jcm15114132

**Published:** 2026-05-27

**Authors:** Maninder Randhawa, Dylan Yu, Anand Rai, Austin Brubaker, Osama Ahmed, Thomas Guyn, Cameron Schaecher, Sara Elzalabany, Abhinav Sood, Jagadeesh K. Kalavakunta, Santhosh K. G. Koshy

**Affiliations:** 1 Department of Internal Medicine, Western Michigan University Homer Stryker M.D. School of Medicine, Kalamazoo, MI 49007, USA; 2 Department of Biomedical Informatics, Western Michigan University Homer Stryker M.D. School of Medicine, Kalamazoo, MI 49007, USA; 3 Department of Cardiology, Beacon Kalamazoo Hospital, Kalamazoo, MI 49048, USA

**Keywords:** acute myocardial infarction, ventricular arrhythmias, racial and ethnic disparities, ventricular tachycardia, ventricular fibrillation, mortality

## Abstract

**Background**: Ventricular arrhythmias (VAs) represent a high-risk complication of acute myocardial infarction (AMI) and are associated with high morbidity and mortality. Racial and ethnic disparities in the management and in-hospital outcomes of AMI with VAs remain incompletely understood. **Methods**: Using the National Inpatient Sample, we conducted a retrospective analysis of hospitalizations in which AMI was complicated by VAs from 2002 to 2022. Hospitalizations were stratified by race/ethnicity including White, Black, Hispanic, and other racial/ethnic groups. Baseline characteristics and in-hospital outcomes were compared across groups. **Results**: We identified 902,398 hospitalizations in which AMI was complicated by VAs, of which 78.2% occurred among White, 9.6% among Black, 6.3% among Hispanic, and 5.9% among patients of other racial/ethnic groups. Compared with White patients, Hispanic (aOR 1.21; CI 1.14–1.28) and patients in other racial/ethnic groups (aOR 1.31; CI 1.24–1.39) had higher odds of in-hospital mortality while Black patients had similar odds. In terms of procedural utilization, Black (aOR 0.65; CI 0.62–0.68), Hispanic (aOR 0.82; CI 0.77–0.86), and other racial/ethnic groups (aOR 0.89; CI 0.85–0.94) all had lower odds of percutaneous coronary intervention (PCI) relative to White patients. Black patients also had lower odds of coronary artery bypass grafting (CABG) (aOR 0.69; CI 0.64–0.74) and implantable cardioverter-defibrillator (ICD) insertion (aOR 0.84; CI 0.74–0.96) compared with White patients during admission. **Conclusions**: Racial and ethnic disparities exist in the prevalence, management, and in-hospital outcomes of AMI complicated by VAs. Further efforts are needed to address differences in care in this high-risk population.

## 1. Introduction

Ventricular arrhythmias (VAs), including ventricular tachycardia (VT) and ventricular fibrillation (VF), are among the most severe complications of acute myocardial infarction (AMI) and are associated with high short-term mortality [1]. Although occurring in a subset of patients, the presence of VAs with AMI identifies a distinct high-risk subgroup characterized by electrical instability and increased risk of sudden cardiac death [2,3,4,5]. In an analysis using data from the Global Registry of Acute Coronary Events (GRACE) registry, researchers found a substantially higher in-hospital mortality rate in patients who develop VAs with AMI compared to those who do not (52% vs. 1.6%) [6]. Although racial and ethnic disparities in the management of AMI are well documented, most studies have evaluated the AMI population more broadly and have not focused on the subset of patients who develop VAs, in which clinical course and in-hospital management differ [7,8,9,10,11,12,13]. To address this gap, we examined racial and ethnic disparities in this high-risk population using a nationally representative sample in the United States.

## 2. Materials and Methods

### 2.1. Study Data

Data was obtained from 1 January 2002 to 31 December 2022 using the National Inpatient Sample (NIS), which is part of the Agency for Healthcare Research and Quality’s Healthcare Cost and Utilization Project [14]. The NIS is the largest deidentified all-payer inpatient database in the U.S., representing a 20% stratified sample of all hospital discharges and enabling nationally representative estimates from over 35 million annual hospitalizations. Institutional Review Board approval was not obtained due to the deidentified nature of the database.

### 2.2. Study Design

We conducted a retrospective observational analysis to study baseline characteristics and in-hospital outcomes of AMI hospitalizations complicated by VAs across different racial/ethnic groups. For our analysis, race/ethnicity was classified as White, Black, Hispanic, and others (Asian or Pacific Islander, Native American, Others). Patient hospitalizations with International Classification of Diseases (ICD) 9 and 10 codes for AMI, STEMI, and NSTEMI as primary diagnoses and VAs, including VT and VF, as secondary diagnosis in any field were included in the study (Supplementary Materials). Patient demographics, insurance type, income quartile, hospital characteristics/region, comorbidities, and in-hospital outcomes were extracted.

Multiple subgroup analyses were additionally performed. The “Others” racial/ethnic category was disaggregated into Asian Pacific Islander, Native American, and all other races to evaluate heterogeneity in outcomes within this group. Additional stratified analyses were conducted by AMI subtype, VA subtype, and exclusion of cardiac arrest to assess whether racial and ethnic differences in outcomes varied in these populations.

### 2.3. Study Outcomes

The primary outcome was in-hospital mortality. Secondary outcomes included length of stay, cost of hospitalization, cardiogenic shock, cardiac arrest, mechanical circulatory support use, renal replacement therapy, acute kidney injury, revascularization with percutaneous coronary intervention (PCI) and coronary artery bypass grafting (CABG), and implantable cardioverter-defibrillator (ICD) insertion. Lastly, year-to-year data in AMI hospitalizations with VAs and in-hospital mortality were extracted from the years 2002 to 2022 across all racial/ethnic groups where sample sizes permitted.

### 2.4. Statistical Analysis

Descriptive statistics were recorded for all variables of interest from the years 2002 to 2022. The year 2015 was excluded due to nationwide transition from ICD-9-CM to ICD-10-CM coding. Hospitalizations with missing race/ethnicity data were excluded from the analysis. All analyses were performed using SAS Studio version 3.82 or SAS 9.4 (SAS Institute, Cary, NC, USA), incorporating the NIS survey design and applying updated trend weights to generate nationally representative estimates.

Baseline characteristics were compared using the Chi-squared test for categorical variables and ANOVA or Kruskal–Wallis test for continuous variables. Multivariable regression models were constructed to minimize confounding when assessing outcomes. Multivariable logistic regression was applied for categorical variables whereas multivariable linear regression was applied for numerical variables, with removal of parameters demonstrating multicollinearity. All variables listed in Table 1 were considered for use in the multivariable regression models. The final list of variables used in the multivariable regression models can be found in the Supplementary Materials. *p* values and adjusted odds ratios for all outcomes were derived from these models. *p*-values < 0.05 were considered statistically significant and all tests were 2-sided with a 95% confidence interval (CI).

Hospitalization costs were adjusted to 2022 U.S. dollars using the Consumer Price Index. National hospitalization rates for each racial/ethnic group were calculated per 100,000 adults using population estimates from the U.S. Census Bureau for each study year [15]. Throughout the analysis, all methodological standards recommended for the use of the NIS were followed.

## 3. Results

### 3.1. Clinical Characteristics of AMI Hospitalizations with VAs

From 2002 to 2022, a total of 902,398 weighted AMI hospitalizations with VAs were identified. Of these hospitalizations, White, Black, Hispanic, and other racial/ethnic groups represented 78.2%, 9.6%, 6.3%, and 5.9% of the study population, respectively. White patients on average were older (median age 66 years), predominately male, and were more likely to belong to middle household incomes. White patients had higher prevalence of atrial fibrillation, dyslipidemia, and prior cardiac procedures when compared to Black, Hispanic, and patients in other racial/ethnic groups.

Black patients on average were younger (median age 62), had the highest proportion of female hospitalizations, and had the highest proportion of patients who belonged to the lowest household income quartile. Black patients had the highest number of comorbidities compared to all other groups including congestive heart failure, valvular disease, obesity, dementia, hypertension, neurological disorders, renal failure, malnutrition, substance abuse, deficiency anemia, prior myocardial infarction, and cerebrovascular disease.

Hispanic patients and patients in other racial/ethnic groups both had a median age of 63 years and were predominately male. Hispanic patients were more likely to belong in the lowest household income quartile while patients in other racial/ethnic groups had the highest proportion of patients in the highest household income quartile. Across all races and ethnicities, most patients were Medicare beneficiaries hospitalized at urban teaching hospitals.

In terms of AMI subtype, patients in other racial/ethnic groups had the highest proportion of presentations with ST-elevation myocardial infarction (STEMI) and lowest presentations with non-ST-elevation myocardial infarction (NSTEMI). Black patients had the lowest proportion of STEMI presentations and highest proportions of NSTEMI presentations when admitted for AMI with VAs (Table 1).

### 3.2. In-Hospital Outcomes of AMI Hospitalizations with VAs

In-hospital mortality differed significantly across all races/ethnicities (*p* < 0.001). Hispanic patients (20.2%) and patients in other racial/ethnic groups (20.1%) had the highest in-hospital mortality, while White patients had the lowest (15.4%). In adjusted analysis with White patients as the reference, Hispanic patients (aOR 1.21; 95% CI 1.14–1.28) and patients in other racial/ethnic groups (aOR 1.31; 95% CI 1.24–1.39) had higher odds of in-hospital mortality, whereas Black patients had similar odds (aOR 1.03; 95% CI 0.98–1.08) compared to White patients.

In terms of other in-hospital outcomes, Hispanic patients (aOR 1.10; 95% CI 1.04–1.16) and patients in other racial/ethnic groups (aOR 1.36; 95% CI 1.28–1.44) had higher odds of cardiogenic shock when compared to White patients, while Black patients had lower odds (aOR 0.77; 95% CI 0.73–0.81). Patients in other racial/ethnic groups additionally had higher odds of cardiac arrest (aOR 1.12; 95% CI 1.07–1.19). Black patients (aOR 0.75; 95% CI 0.71–0.79) had lower odds of mechanical circulatory support use while Hispanic patients (aOR 1.13; 95% CI 1.07–1.20) and patients in other racial/ethnic groups (aOR 1.30; 95% CI 1.22–1.38) had higher odds when compared to White patients.

Black patients (aOR 1.28; 95% CI 1.22–1.35), Hispanic patients (aOR 1.08; 95% CI 1.03–1.14), and patients in other racial/ethnic groups (aOR 1.13; 95% CI 1.08–1.20) had higher odds of acute kidney injury when compared to White patients. Similarly, Black patients (aOR 2.04; 95% CI 1.89–2.20), Hispanic patients (aOR 1.80; 95% CI 1.64–1.97), and patients in other racial/ethnic groups (aOR 1.76; 95% CI 1.59–1.95) had higher odds of renal replacement therapy.

**Figure 1:**
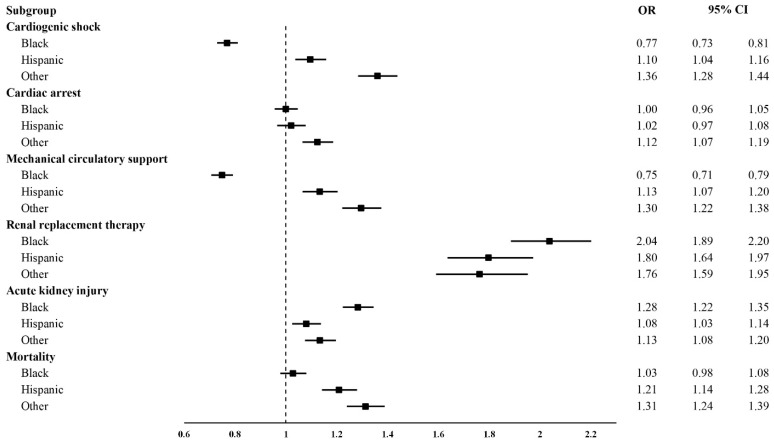
Forest plot depicting adjusted odds ratio and 95% confidence intervals for in-hospital outcomes among all racial/ethnic groups with White patients as the referent. OR = odds ratio; CI = confidence interval.

Length of hospital stay was similar between all cohorts with White patients having the lowest median length of stay at 3.6 days. Patients in other racial/ethnic groups had the highest total cost of hospitalization when compared to White patients, Black patients, and Hispanic patients (*p* < 0.001) (Table 2, Figure 1).

### 3.3. In-Hospital Procedural Utilization

Significant differences in PCI utilization were observed in our analysis (*p* < 0.001). Patients in other racial/ethnic groups had the greatest proportion of PCI (54.8%), followed by White patients (54.4%), Hispanic patients (51.0%), and Black patients (43.7%) (Table 3). After adjusted analysis, Black patients (aOR 0.65; 95% CI 0.62–0.68), Hispanic patients (aOR 0.82; 95% CI 0.77–0.86), and patients in other racial/ethnic groups (aOR 0.89; 95% CI 0.85–0.94) all had lower odds of undergoing PCI compared to White patients.

Similarly, there were significant differences in utilization of CABG across all groups (*p* < 0.001). Patients in other racial/ethnic groups had the greatest proportion of CABG (11.8%), followed by Hispanic patients (11.2%), White patients (10.2%), and Black patients (8.5%). After adjusted analysis with White patients as the reference, Black patients (aOR 0.69; 95% CI 0.64–0.74) had lower odds of undergoing CABG, while Hispanic patients (aOR 0.99; 95% CI 0.92–1.07) had similar odds. Patients in other racial/ethnic groups (aOR 1.14; 95% CI 1.06–1.23) had higher odds compared to White patients to undergo CABG.

**Figure 2:**
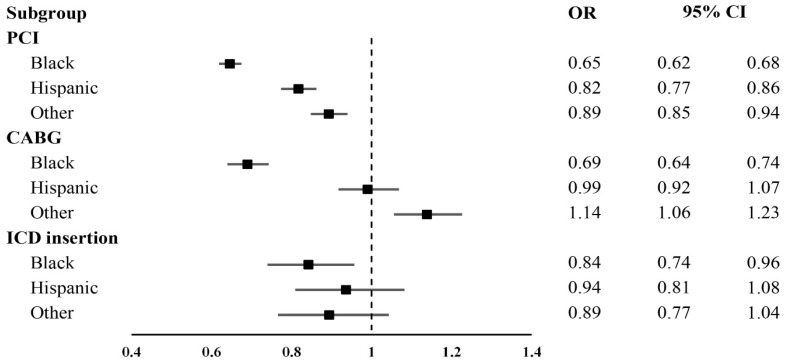
Forest plot depicting adjusted odds ratio and 95% confidence intervals for in-hospital procedural utilization among all racial/ethnic groups with White patients as the referent. PCI = percutaneous coronary intervention; CABG = coronary artery bypass grafting; ICD = implantable cardioverter-defibrillator; OR = odds ratio; CI = confidence interval.

In-hospital ICD insertion was low but differed across all races/ethnicities (*p* = 0.038), with Hispanic patients having the highest proportion (2.0%) and White patients with the lowest (1.7%). After adjusted analysis, Black patients (aOR 0.84; 95% CI 0.74–0.96) had lower odds of ICD insertion while Hispanic patients (aOR 0.94; 95% CI 0.81–1.08) and patients in all other racial/ethnic groups (aOR 0.89; 95% CI 0.77–1.04) had similar odds compared to White patients. (Table 3, Figure 2).

### 3.4. Subgroup Analysis of In-Hospital Outcomes and Procedural Utilization in Other Racial/Ethnic Groups

A subgroup analysis disaggregating other racial/ethnic groups into Asian Pacific Islanders, Native Americans, and all other races was performed. Baseline characteristics for each of these groups can be found in the Supplementary Materials. In adjusted analysis with White patients as the reference, Asian Pacific Islanders (aOR 1.45; 95% CI 1.33–1.58) and all other races (aOR 1.23; 95% CI 1.14–1.33) had higher odds of in-hospital mortality while Native Americans (aOR 1.22; 95% CI 1.00–1.49) had similar odds.

In terms of other in-hospital outcomes, Asian Pacific Islanders (aOR 1.44; 95% CI 1.32–1.57), Native Americans (aOR 1.33; 95% CI 1.10–1.61), and all other races (aOR 1.31; 95% CI 1.21–1.42) had higher odds of cardiogenic shock, while Asian Pacific Islanders (aOR 1.15; 95% CI 1.06–1.25) and all other races (aOR 1.12; 95% CI 1.04–1.20) additionally had higher odds of cardiac arrest. Asian Pacific Islanders (aOR 1.26; 95% CI 1.24–1.49) and all other races (aOR 1.27; 95% CI 1.18–1.37) also had higher odds of mechanical circulatory support use while Native Americans had similar odds (aOR 1.17; 95% CI 0.96–1.41) compared to White patients.

Asian Pacific Islanders (aOR 1.15; 95% CI 1.06–1.25) and all other races (aOR 1.12; 95% CI 1.05–1.21) were also found to have higher odds of acute kidney injury while Native Americans (aOR 1.14; 95% CI 0.94–1.38) had similar odds compared to White patients. Likewise, Asian Pacific Islanders (aOR 2.14; 95% CI 1.86–2.46) and all other races (aOR 1.53; 95% CI 1.32–1.78) had higher odds of renal replacement therapy while Native Americans had similar odds (aOR 1.31; 95% CI 0.91–1.89).

In terms of procedural utilization, Asian Pacific Islanders (aOR 0.80; 95% CI 0.73–0.87) had lower odds of undergoing PCI compared to White patients, while Native Americans (aOR 0.94; 95% CI 0.80–1.11) and all other races (aOR 0.96; 95% CI 0.90–1.02) had similar odds. When analyzing utilization of CABG, Asian Pacific Islanders (aOR 1.18; 95% CI 1.06–1.31) had higher odds compared to White patients while Native Americans (aOR 1.25; 95% CI 0.99–1.57) and all other races (aOR 1.10; 95% CI 0.98–1.22) had similar odds. No significant differences were found in utilization of in-hospital ICD among Asian Pacific Islanders (aOR 0.94; 95% CI 0.75–1.18), Native Americans (aOR 1.08; 95% CI 0.67–1.77), and all other races (aOR 0.83; 95% CI 0.67–1.03) compared to White patients (Table 4).

### 3.5. Subgroup Analysis of Hospitalizations Stratified by AMI Subtype, VA Subtype, and Cardiac Arrest

When analyzing STEMI hospitalizations with VAs, Black patients (aOR 1.17; 95% CI 1.09–1.26), Hispanic patients (aOR 1.22; 95% CI 1.14–1.32), Asian Pacific Islanders (aOR 1.31; 95% CI 1.17–1.46), and all other races (aOR 1.22; 95% CI 1.11–1.34) had higher odds of in-hospital mortality compared to White patients. Native Americans (aOR 1.14; 95% CI 0.88–1.48) had similar odds after adjusted analysis. In terms of coronary revascularization, Black patients (aOR 0.70; 95% CI 0.66–0.75), Hispanic patients (aOR 0.85; 95% CI 0.79–0.91), Asian Pacific Islanders (aOR 0.76; 95% CI 0.69–0.85), and all other races (aOR 0.91; 95% CI 0.83–0.99) had lower odds of undergoing PCI compared to White patients while Native Americans (aOR 0.94; 95% CI 0.74–1.19) had similar odds. Black patients (aOR 0.71; 95% CI 0.63–0.79) also had lower odds of undergoing CABG while all other groups had similar odds to White patients.

For NSTEMI hospitalizations with VAs, Hispanic patients (aOR 1.17; 95% CI 1.07–1.28), Asian Pacific Islanders (aOR 1.66; 95% CI 1.46–1.90), Native Americans (aOR 1.35; 95% CI 1.01–1.80), and all other races (aOR 1.24; 95% CI 1.10–1.40) had higher odds of in-hospital mortality compared to White patients, while Black patients (aOR 0.86; 95% CI 0.80–0.93) had lower odds. In terms of coronary revascularization, Black patients (aOR 0.60; 95% CI 0.56–0.63), Hispanic patients (aOR 0.77; 95% CI 0.71–0.83), and Asian Pacific Islanders (aOR 0.82; 95% CI 0.72–0.93) had lower odds of undergoing PCI compared to White patients. Black patients also had lower odds of undergoing CABG (aOR 0.68; 95% CI 0.62–0.74) and in-hospital ICD insertion (aOR 0.81; 95% CI 0.69–0.94) compared to White patients.

When analyzing VT hospitalizations alone, Hispanic patients (aOR 1.22; 95% CI 1.12–1.33), Asian Pacific Islanders (aOR 1.71; 95% CI 1.51–1.94), Native Americans (aOR 1.40; 95% CI 1.04–1.88), and all other races (aOR 1.28; 95% CI 1.14–1.44) had higher odds of in-hospital mortality while Black patients (aOR 0.95; 95% CI 0.89–1.03) had similar odds compared to White patients. For VF hospitalizations, Black patients (aOR 1.20; 95% CI 1.10–1.31), Hispanic patients (aOR 1.26; 95% CI 1.15–1.38), and all other races (aOR 1.18; 95% CI 1.04–1.34) had higher odds of in-hospital mortality compared to White patients while Asian Pacific Islanders and Native Americans had similar odds (Table 5).

In an analysis excluding patients with cardiac arrest, Hispanic patients (aOR 1.19; 95% CI 1.10–1.28), Asian Pacific Islanders (aOR 1.51; 95% CI 1.35–1.69), and all other races (aOR 1.19; 95% CI 1.07–1.31) had higher odds of in-hospital mortality while Black patients (aOR 0.94; 95% CI 0.88–1.00) and Native Americans (aOR 1.26; 95% CI 0.97–1.63) had similar odds compared to White patients (Table 6).

### 3.6. Temporal Changes in AMI Hospitalizations with VAs and In-Hospital Mortality

**Figure 3:**
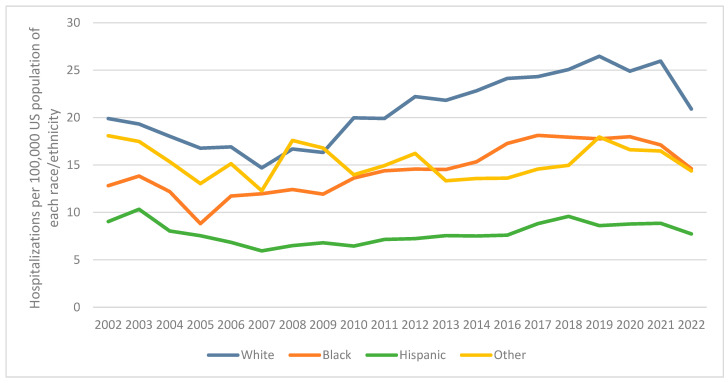
Trends in racial/ethnic differences of hospitalizations with a primary diagnosis of acute myocardial infarction and secondary diagnoses of ventricular tachycardia/fibrillation per 100,000 of the US population of each racial/ethnic group > 18 years old. *p*-value < 0.001.

Among AMI hospitalizations with VAs per 100,000 of the U.S. population of each race/ethnicity, White patients had the highest number of hospitalizations for the majority of the study period, followed by patients in other racial/ethnic groups, Black patients, and Hispanic patients. White patients (20 to 21 per 100,000), Black patients (13 to 15 per 100,000), and Hispanic patients (9 to 8 per 100,000) had relatively stable hospitalization rates during the study period with slight decline in early study years followed by a gradual increase. Patients in other racial/ethnic groups overall had a slight decrease in hospitalization rates during the study period with year-to-year fluctuations observed (18 to 14 per 100,000) (Figure 3).

**Figure 4:**
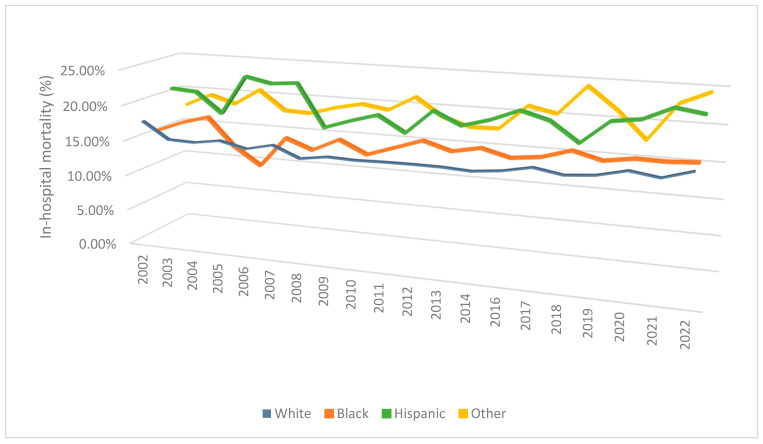
Trends in in-hospital mortality among all races/ethnicities with a primary diagnosis of acute myocardial infarction and secondary diagnoses of ventricular tachycardia/fibrillation >18 years old. *p*-value < 0.001.

Over the same period, in-hospital mortality remained relatively stable in White patients, from 17.6% in 2002 to 17.0% in 2022. In contrast, in-hospital mortality slightly increased in Black patients (15.5% to 17.1%), Hispanic patients (20.8% to 22.4%), and substantially increased in patients of other racial/ethnic groups (17.8% to 24.5%) during the study period (Figure 4). Due to sample size limitations, year-to-year subgroup analysis of patients in other racial/ethnic groups could not be performed.

## 4. Discussion

To our knowledge, this is the first nationally representative study to specifically examine racial and ethnic disparities in patients hospitalized with AMI complicated by VAs. While prior studies have analyzed disparities in AMI broadly, there remains a critical gap in understanding outcomes in the high-risk subgroup where ischemic injury and electrical instability coexist. Several findings emerge from our study. First, Hispanic patients, Asian Pacific Islanders, and patients in all other races experienced higher in-hospital mortality compared to White patients, while Black patients and Native Americans had similar odds after adjusted analysis. Second, disparities in the use of invasive cardiac procedures were observed, with lower utilization of PCI across nearly all non-White patients and lower odds of CABG and ICD insertion among Black patients. Third, Hispanic patients, Asian Pacific Islanders, and all other races had higher odds of cardiogenic shock and mechanical circulatory support use, while most non-White patients had higher odds of acute kidney injury and renal replacement therapy during admission. Finally, in-hospital mortality has slightly increased in Black and Hispanic patients with a more substantial increase observed for patients in other racial/ethnic groups over the past two decades.

Most prior studies analyzing AMI hospitalizations more broadly have not found significant differences in short-term or in-hospital mortality across racial and ethnic groups after adjustment for comorbidities and socioeconomic factors [7,8,9,10,11,12]. In a one-year multi-state analysis, however, Kim et al. reported higher in-hospital mortality among Hispanic and Asian patients compared with non-Hispanic Whites, which aligns with our findings [13]. When focusing specifically on VAs, data regarding racial disparities is limited. In an analysis of two-year hospitalization data for VAs in California, Alexander et al. did not identify significant differences in in-hospital mortality across different racial groups [16]. This suggests that mortality differences may become more apparent when analyzing nationwide data and when specifically focusing on ischemic VAs.

In our study, the higher in-hospital mortality observed among Hispanic patients and those in other racial/ethnic groups is likely due to multiple factors. First, both groups had higher odds of cardiogenic shock, and patients in other racial/ethnic groups additionally had higher odds of cardiac arrest, both of which are strong predictors of in-hospital mortality. Furthermore, both groups had a higher proportion of STEMI presentations, which are associated with greater hemodynamic instability and propagation of malignant VAs [17]. Notably, racial/ethnic disparities in mortality persisted across multiple sensitivity analyses in our study. When stratified by AMI subtype, Hispanic patients and nearly all patients in other racial/ethnic groups continued to experience higher in-hospital mortality compared with White patients. Similar findings were observed in analyses limited to patients with VT alone and excluding cardiac arrest presentations, suggesting that differences in AMI and VA subtype do not fully account for the observed differences in mortality across racial/ethnic groups. Prior literature has suggested limited healthcare access, language barriers, suboptimal control of chronic comorbidities, and delayed presentations as contributors to poor cardiovascular outcomes among Hispanic, Asian Pacific Islanders, and Native Hawaiian populations in the United States [18,19,20,21,22,23]. Thus, these broader structural factors may additionally explain the in-hospital mortality disparities observed in these groups in our analysis.

Interestingly, Black patients had similar adjusted in-hospital mortality as White patients despite having a higher comorbidity burden. This may be explained in part by the higher proportion of NSTEMI presentations in Black patients, which are often observed in patients with greater cardiovascular comorbidities [24,25,26]. Compared with STEMI, NSTEMI presentations are typically associated with smaller infarct size and less hemodynamic compromise with lower short-term mortality [27,28,29,30]. However, this pattern was not consistent across more severe clinical presentations in this population. When stratified by AMI subtype, Black patients presenting with STEMI had higher in-hospital mortality compared with White patients. A similar finding was observed when stratified by VA subtype, where Black patients with VF also experienced higher in-hospital mortality. This finding suggests that although overall mortality is similar in the full AMI and VA cohort, disparities are more apparent among Black patients with higher-risk presentations characterized by greater hemodynamic instability and greater reliance on time-sensitive interventions [31].

In terms of procedural utilization, ICD insertion was low during index admission across all racial/ethnic groups, consistent with current guidelines in management of VAs following AMI and a prior analysis of sex-related disparities in this population [32,33,34]. Although ICD insertion is indicated for secondary prevention in appropriately selected patients, Black patients in our analysis had lower adjusted odds of in-hospital insertion compared with White patients. Although statistically significant, this finding should be interpreted with reservation as the NIS does not capture key determinants of ICD eligibility after AMI including arrhythmia timing and left ventricular function. Further studies with more granular clinical data are needed to better characterize this finding and whether this reflects appropriate clinical management.

Black patients also had lower odds of undergoing CABG, and nearly all non-White patients had lower odds of undergoing PCI in our analysis regardless of AMI subtype. In the setting of AMI complicated by VAs, coronary revascularization is particularly important as it reduces ongoing ischemia and may limit further arrhythmogenic scar formation [35,36]. These findings are consistent with prior literature demonstrating persistent underuse of coronary revascularization among racial and ethnic minority populations [37,38,39]. Multiple factors cited in the literature may have led to these findings, including proximity to PCI-capable facilities, implicit bias among healthcare workers, and patient distrust of the medical system [40,41,42]. Additionally, the higher prevalence of renal dysfunction among non-White patients in our study may have further contributed to the lower use of contrast-based procedures. Ultimately, targeted strategies are needed to address these gaps in care and ensure equitable delivery of guideline-directed therapies in this high-risk population.

Finally, temporal trends revealed a slight increase in in-hospital mortality among Black and Hispanic patients with a more substantial increase in other racial/ethnic groups over the past two decades. These findings may reflect the increasing burden of cardiovascular disease among Black, Hispanic, Asian, and multiracial patients as well as persistent underutilization of guideline-directed therapies over time [43,44,45]. Broader structural and societal factors, including socioeconomic barriers and differences in health literacy, should also be considered in influencing outcome disparities [46,47]. Particular attention is warranted for patients in other racial/ethnic groups, as the factors contributing to their markedly higher increase in in-hospital mortality remain unclear relative to Black and Hispanic populations.

### Limitations

This study has several important limitations. First, the NIS is an administrative database, and coding errors or misclassifications related to ICD-9 and ICD-10 codes may affect the accuracy of identifying AMI and VAs. Second, the transition from ICD-9 to ICD-10 in 2015 may have introduced inconsistencies in coding, and STEMI versus NSTEMI differentiation was limited prior to October 2005. Additionally, NSTEMI presentations may include type 2 AMI presentations due to ambiguity of coding. Third, the NIS captures hospitalizations rather than unique patients, preventing longitudinal patient-level analyses. Fourth, the database lacks data including laboratory values, infarct size, left ventricular function, coronary anatomy, and medication use, which could influence risk adjustment and outcomes. Fifth, differentiation between sustained and non-sustained VAs could not be made due to limitations of ICD-9 and ICD-10 coding, which may introduce important differences in outcomes given that these entities differ in clinical severity, management, and prognosis. Sixth, long-term outcomes beyond hospital discharge, including recurrent arrhythmias, ICD insertion post-discharge, and mortality, could not be assessed. Seventh, the NIS does not capture variables such as door-to-balloon time or angiographic data which may have influenced the in-hospital outcomes seen in our study. Eighth, race and ethnicity are reported at the hospital level and may be subject to misclassification, and the other racial/ethnic group represents a heterogeneous population which could not fully be stratified in trend analysis due to sample size limitation and data reporting guidelines suggested by HCUP. Ninth, although AMI was listed as the primary diagnosis and VAs as secondary diagnoses in our analysis, the NIS does not fully allow determination of the temporal relationship between these events, which may have occurred prior to admission, during resuscitation, or following coronary revascularization, and remains prone to coding errors inherent to administrative databases. Finally, the large sample size may have resulted in statistically significant outcomes which may not be clinically significant. Despite these limitations, this study provides valuable insight into disparities and in-hospital outcomes in this high-risk population.

## 5. Conclusions

In summary, we identified persistent racial and ethnic disparities in the in-hospital management and outcomes in patients hospitalized for AMI complicated by VAs. Hispanic patients, Asian Pacific Islanders, and all other races experienced higher in-hospital mortality and greater prevalence of cardiogenic shock along with other complications, while nearly all non-White patients had lower use of guideline-directed therapies during admission. Over the past two decades, all non-White patients have had an increase in-hospital mortality which was most pronounced in patients belonging in other racial/ethnic groups. These findings highlight important gaps in the equitable delivery of advanced cardiovascular care within a high-risk population and underscore the need for targeted, system-level interventions to improve outcomes across all patients regardless of race and ethnicity.

## Figures and Tables

**Table 1:** Baseline characteristics for patients admitted for acute myocardial infarction with ventricular arrhythmias stratified by race and ethnicity.

	White	Black	Hispanic	Other	Total	*p*-Value
Demographics						
Sample Size	706,090 (78.2%)	86,494 (9.6%)	56,983 (6.3%)	52,830 (5.9%)	902,398 (100.0%)	
Age (Median, IQR)	66 (56.3–76.1)	62 (52.6–71.6)	63 (53.7–72.7)	63 (53.6–72.9)	65 (55.7–75.4)	<0.001
Gender						<0.001
Male	513,714 (72.8%)	55,311 (64.0%)	42,665 (74.9%)	40,199 (76.1%)	651,889 (72.2%)	
Female	192,317 (27.2%)	31,178 (36.0%)	14,318 (25.1%)	12,626 (23.9%)	250,439 (27.8%)	
Insurance type						<0.001
Medicare	381,712 (54.1%)	41,668 (48.3%)	25,087 (44.1%)	21,921 (41.5%)	470,388 (52.2%)	
Medicaid	40,919 (5.8%)	11,935 (13.8%)	8597 (15.1%)	7201 (13.6%)	68,652 (7.6%)	
Private Insurance	221,611 (31.4%)	21,849 (25.3%)	14,701 (25.8%)	17,552 (33.3%)	275,714 (30.6%)	
Self-Pay	36,590 (5.2%)	7382 (8.6%)	6050 (10.6%)	4199 (8.0%)	54,221 (6.0%)	
No Charge/Other	24,141 (3.4%)	3501 (4.1%)	2497 (4.4%)	1902 (3.6%)	32,041 (3.6%)	
Income quartile						<0.001
0–25th	156,012 (23.7%)	40,820 (50.0%)	19,601 (36.7%)	10,088 (20.4%)	226,521 (26.9%)	
26–50th	180,408 (27.4%)	18,214 (22.3%)	12,793 (24.0%)	10,446 (21.1%)	221,861 (26.4%)	
51–75th	168,055 (25.6%)	13,124 (16.1%)	12,504 (23.4%)	12,595 (25.5%)	206,278 (24.5%)	
76–100th	152,824 (23.3%)	9429 (11.6%)	8467 (15.9%)	16,325 (33.0%)	187,045 (22.2%)	
Hospital location						<0.001
Rural	49,415 (7.0%)	3086 (3.6%)	1458 (2.6%)	1611 (3.1%)	55,570 (6.2%)	
Urban Nonteaching	236,504 (33.6%)	21,250 (24.6%)	19,398 (34.1%)	15,701 (29.9%)	292,854 (32.5%)	
Urban Teaching	418,549 (59.4%)	61,902 (71.8%)	36,064 (63.4%)	35,184 (67.0%)	551,700 (61.3%)	
Region of hospital						<0.001
Northeast	153,055 (21.7%)	13,378 (15.5%)	9404 (16.5%)	11,532 (21.8%)	187,369 (20.8%)	
Midwest	155,108 (22.0%)	16,070 (18.6%)	3055 (5.4%)	7046 (13.3%)	181,280 (20.1%)	
South	278,329 (39.4%)	48,612 (56.2%)	24,040 (42.2%)	15,871 (30.0%)	366,852 (40.7%)	
West	119,599 (16.9%)	8433 (9.7%)	20,484 (35.9%)	18,380 (34.8%)	166,896 (18.5%)	
Comorbidities						
Congestive heart failure	160,756 (22.8%)	25,832 (29.9%)	16,953 (29.8%)	15,137 (28.7%)	218,677 (24.2%)	<0.001
Valvular disease	43,461 (6.2%)	6295 (7.3%)	3872 (6.8%)	3296 (6.2%)	56,924 (6.3%)	<0.001
Chronic pulmonary disease	139,177 (19.7%)	15,310 (17.7%)	8048 (14.1%)	6849 (13.0%)	169,383 (18.8%)	<0.001
Obesity	96,556 (13.7%)	13,478 (15.6%)	8507 (14.9%)	5473 (10.4%)	124,014 (13.7%)	<0.001
Dementia	15,115 (2.1%)	2466 (2.9%)	1415 (2.5%)	961.9 (1.8%)	19,959 (2.2%)	<0.001
Dyslipidemia	377,470 (53.5%)	42,633 (49.3%)	29,446 (51.7%)	27,616 (52.3%)	477,165 (52.9%)	<0.001
Diabetes mellitus	179,066 (25.4%)	30,072 (34.8%)	23,218 (40.7%)	18,139 (34.3%)	250,495 (27.8%)	<0.001
Hypertension	420,987 (59.6%)	62,179 (71.9%)	36,966 (64.9%)	32,457 (61.4%)	552,590 (61.2%)	<0.001
Liver disease	14,008 (2.0%)	2648 (3.1%)	1817 (3.2%)	1494 (2.8%)	19,966 (2.2%)	<0.001
Neurological disorders	59,876 (8.5%)	9322 (10.8%)	6056 (10.6%)	5313 (10.1%)	80,568 (8.9%)	<0.001
Peripheral vascular disease	72,082 (10.2%)	8755 (10.1%)	5040 (8.8%)	4436 (8.4%)	90,312 (10.0%)	<0.001
Renal failure	99,319 (14.1%)	21,507 (24.9%)	10,871 (19.1%)	8824 (16.7%)	140,519 (15.6%)	<0.001
Malnutrition	20,084 (2.8%)	3609 (4.2%)	1918 (3.4%)	1948 (3.7%)	27,559 (3.1%)	<0.001
Atrial fibrillation	160,477 (22.7%)	14,545 (16.8%)	10,662 (18.7%)	10,196 (19.3%)	195,880 (21.7%)	<0.001
History of malignancy	21,043 (3.0%)	2388 (2.8%)	1128 (2.0%)	1107 (2.1%)	25,666 (2.8%)	<0.001
Coagulopathy	52,383 (7.4%)	7183 (8.3%)	5694 (10.0%)	5851 (11.1%)	71,110 (7.9%)	<0.001
Drug abuse	14,354 (2.0%)	5966 (6.9%)	1614 (2.8%)	1040 (2.0%)	22,974 (2.5%)	<0.001
Alcohol abuse	25,831 (3.7%)	4451 (5.1%)	2279 (4.0%)	1537 (2.9%)	34,098 (3.8%)	<0.001
Nicotine dependence	182,881 (25.9%)	23,446 (27.1%)	11,530 (20.2%)	11,273 (21.3%)	229,131 (25.4%)	<0.001
Deficiency anemia	84,459 (12.0%)	16,738 (19.4%)	9711 (17.0%)	8209 (15.5%)	119,117 (13.2%)	<0.001
Hypothyroidism	56,726 (8.0%)	3429 (4.0%)	3182 (5.6%)	2771 (5.2%)	66,107 (7.3%)	<0.001
Previous PCI	82,643 (11.7%)	10,081 (11.7%)	6431 (11.3%)	5600 (10.6%)	104,755 (11.6%)	0.012
Previous CABG	78,854 (11.2%)	6473 (7.5%)	4962 (8.7%)	4369 (8.3%)	94,658 (10.5%)	<0.001
Previous MI	87,796 (12.4%)	11,190 (12.9%)	6614 (11.6%)	5809 (11.0%)	111,408 (12.3%)	<0.001
Cerebrovascular disease	49,955 (7.1%)	10,379 (12.0%)	4914 (8.6%)	4354 (8.2%)	69,602 (7.7%)	<0.001
Presence of heart valve replacement	7941 (1.1%)	782.5 (0.9%)	499.4 (0.9%)	408.1 (0.8%)	9631 (1.1%)	<0.001
Presence of cardiac pacemaker	14,180 (2.0%)	1492 (1.7%)	937.2 (1.6%)	807.1 (1.5%)	17,417 (1.9%)	<0.001
Clinical presentation						<0.001
STEMI	400,796 (56.8%)	40,042 (46.3%)	32,517 (57.1%)	31,561 (59.7%)	504,915 (56.0%)	
NSTEMI	306,285 (43.4%)	46,444 (53.7%)	24,467 (42.9%)	21,317 (40.3%)	398,513 (44.2%)	

Values are *n* (%) unless otherwise indicated. MI = Myocardial infarction; STEMI = ST-elevation myocardial infarction; NSTEMI = Non-ST-elevation myocardial infarction; PCI = Percutaneous coronary intervention; CABG = Coronary artery bypass grafting.

**Table 2:** In-hospital outcomes for patients admitted for acute myocardial infarction with ventricular arrhythmias stratified by race and ethnicity.

	White	Black	Hispanic	Other	*p*-Value
Cardiogenic Shock	134,487 (19.0%)	14,607 (16.9%)	13,870 (24.3%)	14,613 (27.7%)	<0.001
Cardiac Arrest	138,305 (19.6%)	17,293 (20.0%)	12,489 (21.9%)	12,216 (23.1%)	<0.001
Mechanical circulatory support	108,864 (15.4%)	11,071 (12.8%)	11,139 (19.5%)	11,840 (22.4%)	<0.001
Renal replacement therapy	19,772 (2.8%)	6377 (7.4%)	3793 (6.7%)	3222 (6.1%)	<0.001
Acute kidney injury	151,827 (21.5%)	25,006 (28.9%)	15,418 (27.1%)	14,063 (26.6%)	<0.001
Mortality	108,414 (15.4%)	13,935 (16.1%)	11,516 (20.2%)	10,621 (20.1%)	<0.001
Length of stay	3.6 (1.8–7.6)	4.1 (2.0–8.7)	4.0 (1.9–8.8)	3.7 (1.8–8.7)	<0.001
Cost of hospitalization (USD 2022)	27,509 (17,506–47,924)	25,890 (15,526–47,655)	31,145 (19,008–57,538)	32,721 (19,868–61,311)	<0.001

Values are *n* (%) unless otherwise indicated.

**Table 3:** Utilization of in-hospital procedures for patients admitted for acute myocardial infarction with ventricular arrhythmias stratified by race and ethnicity.

	White	Black	Hispanic	Other	*p*-Value
PCI	384,357 (54.4%)	37,805 (43.7%)	29,074 (51.0%)	28,970 (54.8%)	<0.001
CABG	72,037 (10.2%)	7312 (8.5%)	6360 (11.2%)	6259 (11.8%)	<0.001
ICD insertion	11,659 (1.7%)	1667 (1.9%)	1119 (2.0%)	939.3 (1.8%)	0.038

Values are *n* (%) unless otherwise indicated. PCI = Percutaneous coronary intervention; CABG = Coronary artery bypass grafting; ICD = Implantable cardioverter-defibrillator.

**Table 4:** In-hospital outcomes and procedural utilization among Asian Pacific Islanders, Native Americans, and all other races when hospitalized for acute myocardial infarction complicated by ventricular arrhythmias.

	Asian Pacific Islander	Native American	All Other Races
Cardiogenic shock	1.44 (1.32–1.57)	1.33 (1.10–1.61)	1.31 (1.21–1.42)
Cardiac arrest	1.15 (1.06–1.25)	1.02 (0.84–1.25)	1.12 (1.04–1.20)
Mechanical circulatory support	1.36 (1.24–1.49)	1.17 (0.96–1.41)	1.27 (1.18–1.37)
Renal replacement therapy	2.14 (1.86–2.46)	1.31 (0.91–1.89)	1.53 (1.32–1.78)
Acute kidney injury	1.15 (1.06–1.25)	1.14 (0.94–1.38)	1.12 (1.05–1.21)
Mortality	1.45 (1.33–1.58)	1.22 (1.00–1.49)	1.23 (1.14–1.33)
PCI	0.80 (0.73–0.87)	0.94 (0.80–1.12)	0.96 (0.90–1.02)
CABG	1.18 (1.06–1.31)	1.25 (0.99–1.57)	1.10 (0.98–1.22)
ICD insertion	0.94 (0.75–1.18)	1.08 (0.67–1.77)	0.83 (0.67–1.03)

Values are aOR (95% CI) with White patients as the reference. PCI = Percutaneous coronary intervention; CABG = Coronary artery bypass grafting; ICD = Implantable cardioverter-defibrillator; aOR = adjusted odds ratio; CI = confidence interval.

**Table 5:** In-hospital outcomes of acute myocardial infarction complicated by ventricular arrhythmias stratified by acute myocardial infarction subtype.

STEMI		Black	Hispanic	Asian Pacific Islander	Native American	All Other Races	*p*-Value
	PCI	0.70 (0.66–0.75)	0.85 (0.79–0.91)	0.76 (0.69–0.85)	0.94 (0.74–1.19)	0.91 (0.83–0.99)	<0.001
	CABG	0.71 (0.63–0.79)	0.94 (0.83–1.06)	1.12 (0.94–1.33)	1.40 (1.00–1.97)	1.12 (0.96–1.31)	<0.001
	ICD insertion	0.90 (0.72–1.13)	0.87 (0.68–1.10)	0.96 (0.67–1.37)	NR ^a^	NR ^a^	NR ^a^
	Mortality	1.17 (1.09–1.26)	1.22 (1.14–1.32)	1.31 (1.17–1.46)	1.14 (0.88–1.48)	1.22 (1.11–1.34)	<0.001
NSTEMI	PCI	0.60 (0.56–0.63)	0.77 (0.72–0.83)	0.82 (0.72–0.93)	0.94 (0.75–1.18)	1.02 (0.93–1.12)	<0.001
	CABG	0.68 (0.62–0.74)	1.03 (0.94–1.13)	1.24 (1.07–1.44)	1.18 (0.88–1.57)	1.10 (0.97–1.24)	<0.001
	ICD insertion	0.81 (0.69–0.94)	0.96 (0.80–1.15)	0.92 (0.68–1.24)	NR ^a^	NR ^a^	NR ^a^
	Mortality	0.86 (0.80–0.93)	1.17 (1.07–1.28)	1.66 (1.46–1.90)	1.35 (1.01–1.80)	1.24 (1.10–1.40)	<0.001

Values are aOR (95% CI) with White patients as the reference. STEMI = ST-elevation myocardial infarction; NSTEMI = Non-ST-elevation myocardial infarction; PCI = Percutaneous coronary intervention; CABG = Coronary artery bypass grafting; ICD = Implantable cardioverter-defibrillator; aOR = adjusted odds ratio; CI = confidence interval. ^a^ *n* < 11 data are not reported (NR) according to HCUP recommendations.

**Table 6:** In-hospital outcomes of acute myocardial infarction complicated by ventricular arrhythmias stratified by ventricular arrhythmia subtype and exclusion of cardiac arrest.

	Black	Hispanic	Asian Pac.	Native American	All Other Races
VT	0.95 (0.89–1.03)	1.22 (1.12–1.33)	1.71 (1.51–1.94)	1.40 (1.04–1.88)	1.28 (1.14–1.44)
VF	1.20 (1.10–1.31)	1.26 (1.15–1.38)	1.11 (0.96–1.28)	0.92 (0.67–1.28)	1.18 (1.04–1.34)
Excluding cardiac arrest	0.94 (0.88–1.00)	1.19 (1.10–1.28)	1.51 (1.35–1.69)	1.26 (0.97–1.63)	1.19 (1.07–1.31)

Values are aOR (95% CI) with White patients as the reference. VT = Ventricular tachycardia; VF = Ventricular fibrillation; aOR = adjusted odds ratio; CI = confidence interval.

## Data Availability

Data used in the study was obtained using the National Inpatient Sample. Please see Supplementary Materials for all data used in the study.

## Supplementary Materials

The following supporting information can be downloaded at: https://www.mdpi.com/article/10.3390/jcm15114132/s1.

## Abbreviations

The following abbreviations are used in this manuscript:

AMI	Acute myocardial infarction
MI	Myocardial infarction
STEMI	ST-elevation myocardial infarction
NSTEMI	Non-ST-elevation myocardial infarction
VA	Ventricular arrhythmia
VT	Ventricular tachycardia
VF	Ventricular fibrillation
PCI	Percutaneous coronary intervention
CABG	Coronary artery bypass grafting
ICD	Implantable cardioverter-defibrillator
aOR	Adjusted odds ratio
