# Supplementary material for: Racial and Ethnic Disparities in the Management and Outcomes of Acute Myocardial Infarction Complicated by Ventricular Arrhythmias
Source: J Clin Med. 2026 May 27;15(11):4132. doi: 10.3390/jcm15114132 (PMC13257581; doi:10.3390/jcm15114132)
Supplement: Supplementary file 1 [file jcm-15-04132-s001.zip › jcm-4294673-supplementary.pdf]

ICD-9/PCS codes used in the analysis.

**Variables**	**ICD-9/PCS Codes**
Acute myocardial infarction	410.71, 410.41, 410.31, 410.91, 410.11, 410.21, 410.01, 410.81, 410.51, 410.61, 410.00, 410.10, 410.20, 410.30, 410.40, 410.50, 410.60, 410.80, 410.90, 410.70
STEMI	410.81, 410.11, 410.31, 410.91, 410.21, 410.01, 410.51, 410.61, 410.41
NSTEMI	410.71
Cardiogenic shock	785.51, 998.01
Congestive heart failure	428.1, 428.20, 428.22, 428.30, 428.32, 428.40, 428.42, 428.90, 428.0, 398.91, 402.01, 402.11, 402.91, 404.01, 404.11, 404.91, 404.03, 404.13, 404.93
Valvular disease	Elixhauser comorbidity
Chronic pulmonary disease	Elixhauser comorbidity
Obesity	Elixhauser comorbidity
Dementia	290, 290.1, 290.11, 290.12, 290.13, 290.2, 290.21, 290.3, 290.4, 290.41, 290.42, 290.43, 291.2, 292.82, 294.1, 294.11, 294.2, 294.21, 331.82, 331.0, 331.11, 331.6, 331.7, 331.81, 331.83 331.89, 331.9, 046.0, 046.11, 046.19, 046.2, 046.3, 331.19, 333.0
Dyslipidemia	272.1, 272.2, 272.3, 272.4, 272.5, 272.9, 272.0, 272.8
Diabetes Mellitus	Elixhauser comorbidity
Hypertension	Elixhauser comorbidity
Liver disease	Elixhauser comorbidity
Neurological disorders	Elixhauser comorbidity
Peripheral vascular disease	Elixhauser comorbidity
Renal failure	Elixhauser comorbidity
Malnutrition	262, 263.0, 263.1, 263.9
Atrial fibrillation	427.31
History of malignancy	Elixhauser comorbidity
Coagulopathy	Elixhauser comorbidity
Drug abuse	Elixhauser comorbidity
Alcohol abuse	Elixhauser comorbidity
Nicotine dependence	Elixhauser comorbidity
Deficiency anemia	Elixhauser comorbidity
Hypothyroidism	Elixhauser comorbidity
Previous PCI	V45.82
Previous CABG	V45.81, 414.02, 414.03, 414.04, 414.05, 414.07
Previous MI	412
Presence of heart valve replacement	V43.3, V42.2
Presence of cardiac pacemaker	V45.01
Cardiac arrest	427.5
Acute Kidney Injury	584.5, 584.6, 584.7, 584.8, 584.9
Mechanical circulatory support	37.66, 37.68, 37.61, 39.65
Renal Replacement Therapy	39.95
Percutaneous Coronary Intervention	00.66, 36.07, 36.06, 17.55
Coronary Artery Bypass Graft	36.10, 36.11, 36.12, 36.13, 36.14, 36.15, 36.16, 36.17, 36.19
ICD insertion	00.52, 37.95, 00.54, 37.96
Ventricular tachycardia	427.1
Ventricular fibrillation	427.41

ICD-10/PCS codes used in the analysis

**Variables**	**ICD-10/PCS Codes**
Acute myocardial infarction	I21.21, I21.01, I21.11, I21.3, I21.02, I21.19, I21.29, I21.09, I22.0, I22.1, I22.8, I22.9, I21.4, I22.2, I21.9
STEMI	I21.21, I21.01, I21.11, I21.3, I21.02, I21.19, I21.29, I21.09, I22.0, I22.1, I22.8, I22.9
NSTEMI	I21.4, I22.2
Cardiogenic shock	R57.0, T81.11XA
Congestive heart failure	I50.1, I50.20, I50.22, I50.30, I50.32, I50.40, I50.42, I50.810, I50.812, I50.814, I50.82, I50.83, I50.84, I50.89, I50.9, I09.81, I11.0, I13.0, I13.2
Valvular disease	Elixhauser comorbidity
Chronic pulmonary disease	Elixhauser comorbidity
Obesity	Elixhauser comorbidity
Dementia	F01.50, F01.51, F02.80, F02.81, F03.90, F03.91, F10.27, F13.27<br>F13.97, F18.17, F18.27, F18.97, F19.17, F19.27, F19.97, G30, G30.0, G30.1, G30.8, G30.9, G31 , G31.01, G31.09, G31.2, G31.83, G31.1, G31.2, G31.81, G31.82, G31.85, G31.89, G31.9<br>A81.00, A81.01, A81.09, A81.1, A81.2
Dyslipidemia	E78, E78.0, E78.00, E78.01, E78.1, E78.2, E78.3, E78.4, E78.41<br>E78.49, E78.5, E78.6, E78.7, E78.70, E78.71, E78.72, E78.79<br>E78.8, E78.81, E78.89, E78.9
Diabetes Mellitus	Elixhauser comorbidity
Hypertension	Elixhauser comorbidity
Liver disease	Elixhauser comorbidity
Neurological disorders	Elixhauser comorbidity
Peripheral vascular disease	Elixhauser comorbidity
Renal failure	Elixhauser comorbidity
Malnutrition	E43, E44.0, E44.1, E46
Atrial fibrillation	I48.0, I48.1, I48.2, I48.91, I48.11, I48.19, I48.20, I48.21
History of malignancy	Elixhauser comorbidity
Coagulopathy	Elixhauser comorbidity
Drug abuse	Elixhauser comorbidity
Alcohol abuse	Elixhauser comorbidity
Nicotine dependence	Elixhauser comorbidity
Deficiency anemia	Elixhauser comorbidity
Hypothyroidism	Elixhauser comorbidity
Previous PCI	Z95.5, Z98.61
Previous CABG	Z95.1, I25.7, I25.70, I25.700, I25.701, I25.702, I25.708, I25.709<br>I25.71, I25.710, I25.711, I25.712, I25.718, I25.719, I25.72<br>I25.720, I25.721, I25.722, I25.728, I25.729, I25.73, I25.730<br>I25.731, I25.732, I25.738, I25.739, I25.810, I25.812
Previous MI	I25.2
Presence of heart valve replacement	Z95.2, Z95.3, Z95.4
Presence of cardiac pacemaker	Z95.0
Cardiac arrest	I46.2, I46.8, I46.9
Acute Kidney Injury/Failure	N17.0, N17.1, N17.2, N17.8, N17.9, N99.0
Mechanical circulatory support	5A1522F, 5A1522G, 5A15A2F, 5A15A2G, 5A1522H, 5A15223, 5A02210, 5A02110, 02HA3QZ, 02HA0QZ, 5A0211D, 5A0221D, 02HA3RJ, 02HA4RJ, 5A02116, 5A02216, 02HA4RZ, 02HA3RZ, 02HA4QZ
Renal Replacement Therapy	5A1D70Z, 5A1D80Z, 5A1D90Z, 5A1D60Z, 5A1D00Z
Percutaneous Coronary Intervention	027034, 0270346, 027034Z, 027035, 0270356, 027035Z, 027036 , 0270366, 027036Z, 027037, 0270376, 027037Z, 027044, 0270446, 027044Z, 027045, 0270456, 027045Z, 027046, 0270466, 027046Z, 027047, 0270476, 027047Z, 0271346<br>027134Z, 027135, 0271356, 027135Z, 027136, 0271366, 027136Z, 027137, 0271376, 027137Z, 027144, 0271446, 027144Z, 0271456, 027145Z, 0271466, 027146Z, 0271476<br>027147Z, 027234, 0272346, 027234Z, 027235, 0272356, 027235Z<br>027236, 0272366, 027236Z, 027237, 0272376, 027237Z, 027244, 0272446, 027244Z, 027245, 0272456, 027245Z, 027246, 0272466<br>027246Z, 027247, 0272476, 027247Z, 027334, 0273346, 027334Z, 0273356, 027335Z, 0273366, 027336Z, 0273376, 027337Z, 027344, 0273446, 027344Z, 0273456, 027345Z, 0273466, 027346Z, 0273476, 027347Z, 02703D, 02703D6, 02703DZ, 02703E, 02703E6, 02703EZ, 02703F, 02703F6, 02703FZ, 02703G, 02703G6, 02703GZ, 02704D, 02704D6, 02704DZ, 02704E, 02704E6, 02704EZ, 02704F, 02704F6, 02704FZ, 02704G, 02704G6, 02704GZ, 02713D6, 02713DZ, 02713E, 02713E6, 02713EZ, 02713F, 02713F6, 02713FZ, 02713G, 02713G6, 02713GZ, 02714D, 02714D6, 02714DZ, 02714E, 02714E6, 02714EZ, 02714F, 02714F6, 02714FZ , 02714G, 02714G6, 02714GZ, 02723D, 02723D6, 02723DZ, 02723E, 02723E6, 02723EZ, 02723F, 02723F6, 02723FZ, 02723G, 02723G6, 02723GZ, 02724D, 02724D6, 02724DZ, 02724E, 02724E6, 02724EZ, 02724F, 02724F6, 02724FZ, 02724G, 02724G6, 02724GZ, 02733D, 02733D6, 02733DZ<br>02733E, 02733E6, 02733EZ, 02733F, 02733F6, 02733FZ, 02733G, 02733G6, 02733GZ, 02734D, 02734D6, 02734DZ, 02734E, 02734E6, 02734EZ, 02734F, 02734F6, 02734FZ, 02734G, 02734G6,

	02734GZ, 02703Z, 02703Z6, 02703ZZ, 02704Z6, 02704ZZ, 02713Z, 02713Z6, 02713ZZ, 02714Z, 02714Z6, 02714ZZ, 02723Z, 02723Z6, 02723ZZ, 02724Z, 02724Z6, 02724ZZ, 02733Z, 02733Z6, 02733ZZ, 02734Z, 02734Z6, 02734ZZ
Coronary Artery Bypass Graft	02130KW, 02130Z3, 02130Z8, 02130Z9, 02130ZC, 02130ZF, 02130K8, 02130K9, 02130KC, 02130KF, 02130A9, 02130AC, 02130AF, 02130AW, 02130J3, 02130J8, 02130J9, 02130JC, 02130JF, 02130JW, 02130K3, 02120Z8, 02120Z9, 02120ZC, 02120ZF, 0213093, 0213098, 0213099, 021309C, 021309F, 021309W, 02130A3, 02130A8, 02120AW, 02120J3, 02120J8, 02120J9, 02120JC, 02120JF, 02120JW, 02120K3, 02120K8, 02120K9, 02120KC, 02120KF, 02120KW, 02120Z3, 02110Z9<br>02110ZC, 02110ZF, 0212093, 0212098, 0212099, 021209C<br>021209F, 021209W, 02120A3, 02120A8, 02120A9, 02120AC,<br>02120AF, 02110J3, 02110J8, 02110J9, 02110JC, 02110JF, 02110JW, 02110K3, 02110K8, 02110K9, 02110KC, 02110KF<br>02110KW, 02110Z3, 02110Z8, 02100ZC, 02100ZF, 0211093<br>0211098, 0211099, 021109C, 021109F, 021109W, 02110A3<br>02110A8, 02110A9, 02110AC, 02110AF, 02110AW, 02100J3<br>02100K9, 02100KC, 02100KF, 02100KW, 02100Z3, 02100Z8, 02100Z9, 0210093, 0210099, 021009C, 021009F, 021009W, 02100A3, 02100A8, 02100A9, 02100AC, 02100AF, 02100AW
ICD insertion	02H43KZ, 02H60KZ, 02H63KZ, 02H64KZ, 02H70KZ, 02H73KZ, 02H74KZ, 02HK0KZ, 02HK3KZ, 02HK4KZ, 02HL0KZ, 02HL3KZ, 02HL4KZ, 0JH609Z, 0JH608Z, 0JH638Z, 0JH639Z, 0JH63FZ, 02H40KZ, 02HN0KZ, 02HN3KZ, 02H44KZ, 02HN4KZ, 0JH60FZ
Ventricular tachycardia	I47.2
Ventricular fibrillation	I49.01

Adjusted odds ratios of in-hospital outcomes with White patients as reference

	Odds ratio	95% CI
Cardiogenic Shock		
A. Black	0.769	(0.730, 0.811)
B. Hispanic	1.096	(1.037, 1.159)
C. Other	1.360	(1.284, 1.439)
Cardiac Arrest		
A. Black	1.000	(0.956, 1.047)
B. Hispanic	1.021	(0.966, 1.078)
C. Other	1.124	(1.065, 1.186)
Mechanical circulatory support		
A. Black		
B. Hispanic	0.749	(0.707, 0.792)
C. Other	1.133	(1.066, 1.204)
	1.296	(1.222, 1.375)
Renal replacement therapy		
A. Black		
B. Hispanic	2.037	(1.885, 2.201)
C. Other	1.797	(1.636, 1.973)
	1.762	(1.591, 1.952)
Acute kidney injury/failure (AKI)		
A. Black	1.283	(1.224, 1.345)
B. Hispanic	1.080	(1.025, 1.138)
C. Other	1.134	(1.075, 1.197)
PCI		
A. Black	0.646	(0.619, 0.675)
B. Hispanic	0.817	(0.774, 0.862)
C. Other	0.893	(0.848, 0.940)
CABG		
A. Black	0.690	(0.640. 0.743)
B. Hispanic	0.990	(0.917, 1.068)
C. Other	1.138	(1.056, 1.226)
ICD insertion		
A. Black	0.842	(0.740, 0.957)
B. Hispanic	0.936	(0.809, 1.082)
C. Other	0.894	(0.766, 1.043)
Mortality		
A. Black	1.028	(0.978, 1.081)
B. Hispanic	1.209	(1.142, 1.280)
C. Other	1.313	(1.240, 1.389)

Baseline characteristics for patients admitted for acute myocardial infarction with ventricular arrhythmias stratified subgroup analysis in Asian Pacific Islanders, Native Americans, and All Other Races

	Asian Pacific Islander	Native American	All Other Races
Demographics			
Sample Size	19,697	4,224	28,909
Age (Median, IQR)	65 (54.4 – 74.6)	63 (53.5 – 71.3)	62 (53.2 – 72.2)
Gender			
Male	14,873 (75.5%)	3,201 (75.8%)	22,125 (76.5%)
Female	4,824 (24.5%)	1,024 (24.2%)	6,779 (23.5%)
Insurance type			
Medicare	8,320 (42.3%)	2,030 (48.2%)	11,571 (40.1%)
Medicaid	2,719 (13.8%)	595.9 (14.1%)	3,886 (13.5%)
Private Insurance	6,790 (34.5%)	1,026 (24.4%)	9,736 (33.7%)
Self-Pay	1,282 (6.5%)	285.2 (6.8%)	2,632 (9.1%)
No Charge/Other	575.8 (2.9%)	276.2 (6.6%)	1,050 (3.6%)
Income quartile			
0-25th	2,111 (11.3%)	1,652 (41.7%)	6,325 (23.6%)
26-50th	3,380 (18.1%)	1,106 (27.9%)	5,959 (22.3%)
51-75th	5,011 (26.8%)	705.3 (17.8%)	6,878 (25.7%)
76-100th	8,216 (43.9%)	498.6 (12.6%)	7,611 (28.4%)
Hospital location			
Rural	499.4 (2.5%)	391.3 (9.7%)	720.4 (2.5%)
Urban Nonteaching	5,910 (30.1%)	1,134 (28.2%)	8,657 (30.0%)
Urban Teaching	13,206 (67.3%)	2,490 (62.0%)	19,489 (67.5%)
Region of hospital			
Northeast	3,172 (16.1%)	384.8 (9.1%)	7,976 (27.6%)
Midwest	1,615 (8.2%)	878.6 (20.8%)	4,552 (15.7%)
South	3,533 (17.9%)	1,676 (39.7%)	10,662 (36.9%)
West	11,377 (57.8%)	1,285 (30.4%)	5,719 (19.8%)
Comorbidities			
Congestive heart failure	6,231 (31.6%)	1,128 (26.7%)	7,778 (26.9%)
Valvular disease	1,504 (7.6%)	240 (5.7%)	1,552 (5.4%)
Chronic pulmonary disease	2,091 (10.6%)	693.8 (16.4%)	4,064 (14.1%)
Obesity	1,536 (7.8%)	712.8 (16.9%)	3,224 (11.2%)
Dementia	389.3 (2.0%)	64.2 (1.5%)	508.4 (1.8%)
Dyslipidemia	10,771 (54.7%)	2,243 (53.1%)	14,603 (50.5%)
Diabetes mellitus	7,687 (39.0%)	1,472 (34.8%)	8,981 (31.1%)
Hypertension	12,741 (64.7%)	2,490 (59.0%)	17,225 (59.6%)
Liver disease	684.6 (3.5%)	99.5 (2.4%)	709.5 (2.5%)

Neurological disorders	2,209<br>(11.2%)	341.9<br>(8.1%)	2,762<br>(9.6%)
Peripheral vascular disease	1,680<br>(8.5%)	444.5<br>(10.5%)	2,311<br>(8.0%)
Renal failure	4,138<br>(21.0%)	671.3<br>(15.9%)	4,014<br>(13.9%)
Malnutrition	762.4<br>(3.9%)	176<br>(4.2%)	1,010<br>(3.5%)
Atrial fibrillation	4,172<br>(21.2%)	718.8<br>(17.0%)	5,305<br>(18.4%)
History of malignancy	452.6<br>(2.3%)	88.8<br>(2.1%)	565.4<br>(2.0%)
Coagulopathy	2,552<br>(13.0%)	349.8<br>(8.3%)	2,949<br>(10.2%)
Drug abuse	247.3<br>(1.3%)	140.7<br>(3.3%)	651.5<br>(2.3%)
Alcohol abuse	335.9<br>(1.7%)	228.4<br>(5.4%)	972.3<br>(3.4%)
Nicotine dependence	3,089<br>(15.7%)	1,133<br>(26.8%)	7,051<br>(24.4%)
Deficiency anemia	3,789<br>(19.2%)	556.4<br>(13.2%)	3,864<br>(13.4%)
Hypothyroidism	1,016<br>(5.2%)	272.5<br>(6.5%)	1,483<br>(5.1%)
Previous PCI	2,123<br>(10.8%)	480.5<br>(11.4%)	2,996<br>(10.4%)
Previous CABG	1,657<br>(8.4%)	441.4<br>(10.4%)	2,271<br>(7.9%)
Previous MI	2,194<br>(11.1%)	530.8<br>(12.6%)	3,084<br>(10.7%)
Cerebrovascular disease	1,766<br>(9.0%)	383.1<br>(9.1%)	2,205<br>(7.6%)
Presence of heart valve replacement	129<br>(0.7%)		
Presence of cardiac pacemaker	380.7<br>(1.9%)	63.7<br>(1.5%)	362.7<br>(1.3%)
Clinical presentation			
STEMI	11,568<br>(58.7%)	2,245<br>(53.1%)	17,748<br>(61.4%)
NSTEMI	8,159<br>(41.4%)	1,994<br>(47.2%)	11,164<br>(38.6%)

Variables used in the multivariable regression model (Tables 2, 3, 4, and 6; Figures 1 and 2):

Gender
Insurance Type
Income Quartile
Urban vs Rural Hospital
Region of Hospital
Congestive Heart Failure
Chronic Pulmonary Disease
Obesity
Dementia
Dyslipidemia
Diabetes Mellitus
Hypertension
Liver Disease
Peripheral Vascular Disease
Malnutrition
Atrial Fibrillation
History of Malignancy
Coagulopathy
Drug Abuse
Nicotine Dependence
Deficiency Anemia
Hypothyroidism
Previous PCI
Cerebrovascular Disease
Presence of Heart Valve Replacement
Presence of Cardiac Pacemaker
STEMI

Variables used in the multivariable regression model (Table 5):

Age
Gender
Insurance Type
Income Quartile
Urban vs Rural Hospital
Region of Hospital
Congestive Heart Failure
Chronic Pulmonary Disease
Obesity
Dementia
Dyslipidemia
Diabetes Mellitus
Hypertension
Liver Disease
Peripheral Vascular Disease
Malnutrition
Atrial Fibrillation
History of Malignancy
Coagulopathy
Drug Abuse
Nicotine Dependence
Deficiency Anemia
Hypothyroidism
Previous PCI
Cerebrovascular Disease
Presence of Heart Valve Replacement
Presence of Cardiac Pacemaker
STEMI
